# *N*-(4-Chloro­phen­yl)-7-oxabicyclo­[2.2.1]hept-5-ene-2,3-dicarboximide

**DOI:** 10.1107/S1600536810047537

**Published:** 2010-11-20

**Authors:** Jian Li

**Affiliations:** a Department of Chemistry and Chemical Engineering, Weifang University, Weifang 261061, People’s Republic of China

## Abstract

In the title racemic compound, C_14_H_10_ClNO_3_, which contains four stereogenic centres, the cyclo­hexane ring tends towards a boat conformation, while the tetra­hydro­furan and dihydro­furan rings adopt envelope conformations. The dihedral angle between the mean planes of the pyrrolidine-2,5-dione unit and the 4-chloro­phenyl ring is 49.0 (2)°.

## Related literature

For the biological activity of 7-oxa-bicyclo­[2,2,1]hept-5-ene-2,3-dicarb­oxy­lic anhydride, see: Deng & Hu (2007 ▶). For related structures, see: Goh *et al.* (2008 ▶); Hart *et al.* (2004 ▶).
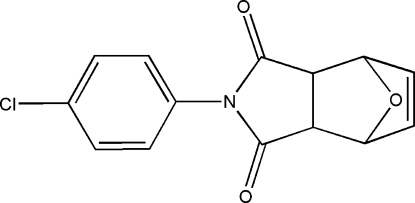


## Experimental

### 

#### Crystal data

C_14_H_10_ClNO_3_
*M* *_r_* = 275.68
Monoclinic, 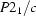

*a* = 10.4946 (11) Å
*b* = 8.2890 (8) Å
*c* = 14.0871 (13) Å
β = 91.538 (1)°
*V* = 1225.0 (2) Å^3^
*Z* = 4
Mo *K*α radiation
μ = 0.31 mm^−1^
*T* = 298 K
0.40 × 0.33 × 0.21 mm

#### Data collection

Bruker SMART CCD area-detector diffractometer
Absorption correction: multi-scan (*SADABS*; Sheldrick, 1996 ▶) *T*_min_ = 0.885, *T*_max_ = 0.937
5907 measured reflections
2156 independent reflections
1466 reflections with *I* > 2σ(*I*)
*R*_int_ = 0.040

#### Refinement

*R*[*F* ^2^ > 2σ(*F* ^2^)] = 0.043
*wR*(*F* ^2^) = 0.113
*S* = 1.07
2156 reflections
172 parameters
H-atom parameters constrained
Δρ_max_ = 0.25 e Å^−3^
Δρ_min_ = −0.33 e Å^−3^

### 

Data collection: *SMART* (Bruker, 1997 ▶); cell refinement: *SAINT* (Bruker, 1997 ▶); data reduction: *SAINT*; program(s) used to solve structure: *SHELXS97* (Sheldrick, 2008 ▶); program(s) used to refine structure: *SHELXL97* (Sheldrick, 2008 ▶); molecular graphics: *SHELXTL* (Sheldrick, 2008 ▶); software used to prepare material for publication: *SHELXTL*.

## Supplementary Material

Crystal structure: contains datablocks global, I. DOI: 10.1107/S1600536810047537/is2633sup1.cif

Structure factors: contains datablocks I. DOI: 10.1107/S1600536810047537/is2633Isup2.hkl

Additional supplementary materials:  crystallographic information; 3D view; checkCIF report

## Figures and Tables

**Fig. 1.**
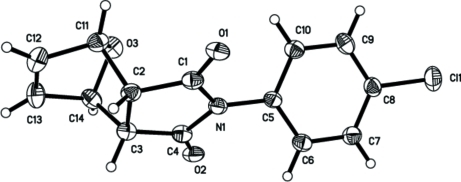
The molecular structure of (I), with the atom-numbering scheme. Displacement ellipsoids are drawn at 30% probability level.

## supplementary crystallographic information

### Comment

7-Oxa-bicyclo[2,2,1]hept-5-ene-2,3-dicarboxylic anhydride has been widely employed in clinical practice, as it is less toxic and much easier to be synthesized (Deng & Hu, 2007). Its derivatives are also pharmacologically active (Hart *et al.*, 2004). In this paper, the structure of the title compound, (I), is reported (Fig. 1). The bond lengths and angles are as expected and comparable to those in the similar compounds (Goh *et al.*, 2008). The dihedral angle between the pyrrolidine-2,5-dione plane and 4-chlorophenyl plane is 49.0 (2)°.

### Experimental

A mixture of *exo*-7-oxa-bicyclo[2,2,1]hept-5-ene-2,3-dicarboxylic anhydride (0.332 g, 2 mmol) and *p*-chloroaniline (0.255 g, 2 mmol) in methanol (5 ml) was stirred for 5 h at room temperature, and then refluxed for 1 h. After cooling, the precipitate was filtered and dried. The crude product of 20 mg was dissolved in methanol of 10 ml. The solution was filtered to remove impurities, and then the filtrate was left for crystallization at room temperature. Single-crystals suitable for X-ray diffraction were obtained by evaporation from the methanol solution after 5 d.

### Refinement

H atoms were initially located from difference maps and then refined in a riding model, with C—H = 0.93 or 0.98 Å and *U*_iso_(H) = 1.2*U*_eq_(C).

### Crystal data

**Table:**

C_14_H_10_ClNO_3_	*F*(000) = 568
*M**_r_* = 275.68	*D*_x_ = 1.495 Mg m^−^^3^
Monoclinic, *P*2_1_/*c*	Mo *K*α radiation, λ = 0.71073 Å
Hall symbol: -P 2ybc	Cell parameters from 1615 reflections
*a* = 10.4946 (11) Å	θ = 2.9–24.8°
*b* = 8.2890 (8) Å	µ = 0.31 mm^−^^1^
*c* = 14.0871 (13) Å	*T* = 298 K
β = 91.538 (1)°	Block, light yellow
*V* = 1225.0 (2) Å^3^	0.40 × 0.33 × 0.21 mm
*Z* = 4	

### Data collection

**Table:**

Bruker SMART CCD area-detector diffractometer	2156 independent reflections
Radiation source: fine-focus sealed tube	1466 reflections with *I* > 2σ(*I*)
graphite	*R*_int_ = 0.040
φ and ω scans	θ_max_ = 25.0°, θ_min_ = 1.9°
Absorption correction: multi-scan (*SADABS*; Sheldrick, 1996)	*h* = −12→12
*T*_min_ = 0.885, *T*_max_ = 0.937	*k* = −9→9
5907 measured reflections	*l* = −13→16

### Refinement

**Table:**

Refinement on *F*^2^	Primary atom site location: structure-invariant direct methods
Least-squares matrix: full	Secondary atom site location: difference Fourier map
*R*[*F*^2^ > 2σ(*F*^2^)] = 0.043	Hydrogen site location: inferred from neighbouring sites
*wR*(*F*^2^) = 0.113	H-atom parameters constrained
*S* = 1.07	*w* = 1/[σ^2^(*F*_o_^2^) + (0.0446*P*)^2^ + 0.4264*P*] where *P* = (*F*_o_^2^ + 2*F*_c_^2^)/3
2156 reflections	(Δ/σ)_max_ < 0.001
172 parameters	Δρ_max_ = 0.25 e Å^−^^3^
0 restraints	Δρ_min_ = −0.33 e Å^−^^3^

### Special details

**Table:**

Geometry. All e.s.d.'s (except the e.s.d. in the dihedral angle between two l.s. planes) are estimated using the full covariance matrix. The cell e.s.d.'s are taken into account individually in the estimation of e.s.d.'s in distances, angles and torsion angles; correlations between e.s.d.'s in cell parameters are only used when they are defined by crystal symmetry. An approximate (isotropic) treatment of cell e.s.d.'s is used for estimating e.s.d.'s involving l.s. planes.
Refinement. Refinement of *F*^2^ against ALL reflections. The weighted *R*-factor *wR* and goodness of fit *S* are based on *F*^2^, conventional *R*-factors *R* are based on *F*, with *F* set to zero for negative *F*^2^. The threshold expression of *F*^2^ > σ(*F*^2^) is used only for calculating *R*-factors(gt) *etc*. and is not relevant to the choice of reflections for refinement. *R*-factors based on *F*^2^ are statistically about twice as large as those based on *F*, and *R*- factors based on ALL data will be even larger.

### Fractional atomic coordinates and isotropic or equivalent isotropic displacement parameters (Å^2^)

**Table:**

	*x*	*y*	*z*	*U*_iso_*/*U*_eq_	
Cl1	1.06821 (7)	0.52842 (11)	0.16059 (6)	0.0601 (3)	
N1	0.58629 (19)	0.2856 (3)	0.31440 (13)	0.0349 (5)	
O1	0.66611 (17)	0.1666 (3)	0.45091 (12)	0.0482 (5)	
O2	0.45183 (18)	0.3870 (3)	0.19755 (13)	0.0556 (6)	
O3	0.37280 (17)	0.4471 (2)	0.44140 (13)	0.0444 (5)	
C1	0.5766 (3)	0.2056 (3)	0.40149 (18)	0.0369 (6)	
C2	0.4383 (2)	0.1821 (3)	0.42057 (17)	0.0361 (6)	
H2	0.4176	0.0697	0.4354	0.043*	
C3	0.3661 (2)	0.2439 (3)	0.33183 (17)	0.0353 (6)	
H3	0.3157	0.1598	0.2994	0.042*	
C4	0.4678 (2)	0.3141 (3)	0.27085 (18)	0.0369 (6)	
C5	0.7041 (2)	0.3409 (3)	0.27702 (17)	0.0325 (6)	
C6	0.7316 (2)	0.3134 (3)	0.18301 (17)	0.0377 (7)	
H6	0.6748	0.2558	0.1442	0.045*	
C7	0.8436 (2)	0.3715 (3)	0.14685 (18)	0.0397 (7)	
H7	0.8623	0.3545	0.0835	0.048*	
C8	0.9268 (2)	0.4544 (3)	0.2053 (2)	0.0407 (7)	
C9	0.9002 (2)	0.4827 (3)	0.2991 (2)	0.0432 (7)	
H9	0.9575	0.5398	0.3377	0.052*	
C10	0.7882 (2)	0.4258 (3)	0.33505 (18)	0.0381 (7)	
H10	0.7692	0.4445	0.3982	0.046*	
C11	0.3888 (3)	0.3030 (4)	0.49633 (18)	0.0427 (7)	
H11	0.4427	0.3138	0.5539	0.051*	
C12	0.2531 (3)	0.2520 (4)	0.5124 (2)	0.0519 (8)	
H12	0.2220	0.1981	0.5648	0.062*	
C13	0.1885 (3)	0.3001 (4)	0.4365 (2)	0.0501 (8)	
H13	0.1015	0.2875	0.4243	0.060*	
C14	0.2830 (2)	0.3796 (3)	0.37332 (19)	0.0432 (7)	
H14	0.2469	0.4559	0.3268	0.052*	

### Atomic displacement parameters (Å^2^)

**Table:**

	*U*^11^	*U*^22^	*U*^33^	*U*^12^	*U*^13^	*U*^23^
Cl1	0.0375 (4)	0.0665 (6)	0.0769 (6)	−0.0031 (4)	0.0100 (4)	0.0018 (4)
N1	0.0347 (12)	0.0361 (14)	0.0336 (12)	−0.0010 (10)	−0.0036 (9)	0.0051 (10)
O1	0.0459 (12)	0.0553 (14)	0.0429 (11)	0.0061 (10)	−0.0086 (9)	0.0132 (10)
O2	0.0503 (12)	0.0730 (16)	0.0430 (12)	0.0006 (11)	−0.0072 (9)	0.0236 (11)
O3	0.0454 (11)	0.0324 (11)	0.0551 (12)	−0.0050 (9)	−0.0040 (9)	−0.0061 (9)
C1	0.0467 (16)	0.0283 (16)	0.0354 (14)	0.0026 (13)	−0.0017 (12)	0.0011 (12)
C2	0.0396 (15)	0.0312 (15)	0.0376 (14)	−0.0008 (12)	0.0009 (12)	0.0058 (12)
C3	0.0364 (15)	0.0312 (15)	0.0380 (14)	−0.0059 (12)	−0.0040 (11)	0.0003 (12)
C4	0.0413 (16)	0.0339 (16)	0.0351 (15)	−0.0025 (13)	−0.0059 (12)	−0.0018 (13)
C5	0.0348 (14)	0.0275 (15)	0.0351 (14)	0.0032 (11)	−0.0015 (11)	0.0025 (11)
C6	0.0389 (15)	0.0378 (17)	0.0362 (15)	−0.0007 (13)	−0.0059 (12)	−0.0006 (13)
C7	0.0443 (16)	0.0386 (17)	0.0364 (15)	0.0044 (14)	0.0039 (12)	−0.0004 (13)
C8	0.0340 (15)	0.0359 (17)	0.0521 (18)	0.0043 (13)	0.0035 (13)	0.0038 (14)
C9	0.0344 (15)	0.0430 (18)	0.0516 (18)	−0.0021 (13)	−0.0069 (13)	−0.0070 (14)
C10	0.0423 (15)	0.0387 (17)	0.0330 (14)	0.0028 (13)	−0.0018 (12)	−0.0036 (12)
C11	0.0461 (17)	0.0462 (18)	0.0355 (15)	−0.0017 (14)	−0.0021 (12)	0.0004 (13)
C12	0.0538 (19)	0.050 (2)	0.0523 (18)	−0.0059 (16)	0.0158 (15)	−0.0042 (16)
C13	0.0371 (16)	0.049 (2)	0.065 (2)	−0.0053 (14)	0.0093 (15)	−0.0124 (16)
C14	0.0392 (15)	0.0390 (17)	0.0510 (17)	0.0016 (14)	−0.0083 (13)	−0.0028 (14)

### Geometric parameters (Å, °)

**Table:**

Cl1—C8	1.740 (3)	C5—C6	1.382 (3)
N1—C4	1.392 (3)	C6—C7	1.380 (3)
N1—C1	1.400 (3)	C6—H6	0.9300
N1—C5	1.432 (3)	C7—C8	1.369 (4)
O1—C1	1.199 (3)	C7—H7	0.9300
O2—C4	1.204 (3)	C8—C9	1.378 (4)
O3—C11	1.431 (3)	C9—C10	1.376 (4)
O3—C14	1.440 (3)	C9—H9	0.9300
C1—C2	1.496 (4)	C10—H10	0.9300
C2—C3	1.533 (3)	C11—C12	1.508 (4)
C2—C11	1.563 (4)	C11—H11	0.9800
C2—H2	0.9800	C12—C13	1.313 (4)
C3—C4	1.505 (4)	C12—H12	0.9300
C3—C14	1.547 (4)	C13—C14	1.503 (4)
C3—H3	0.9800	C13—H13	0.9300
C5—C10	1.380 (3)	C14—H14	0.9800
			
C4—N1—C1	112.4 (2)	C8—C7—H7	120.4
C4—N1—C5	123.6 (2)	C6—C7—H7	120.4
C1—N1—C5	123.9 (2)	C7—C8—C9	121.3 (2)
C11—O3—C14	95.76 (19)	C7—C8—Cl1	119.7 (2)
O1—C1—N1	124.2 (2)	C9—C8—Cl1	118.9 (2)
O1—C1—C2	127.6 (2)	C10—C9—C8	119.5 (3)
N1—C1—C2	108.2 (2)	C10—C9—H9	120.3
C1—C2—C3	105.7 (2)	C8—C9—H9	120.3
C1—C2—C11	112.4 (2)	C9—C10—C5	119.7 (2)
C3—C2—C11	100.1 (2)	C9—C10—H10	120.1
C1—C2—H2	112.6	C5—C10—H10	120.1
C3—C2—H2	112.6	O3—C11—C12	102.6 (2)
C11—C2—H2	112.6	O3—C11—C2	101.68 (19)
C4—C3—C2	104.6 (2)	C12—C11—C2	104.8 (2)
C4—C3—C14	110.5 (2)	O3—C11—H11	115.3
C2—C3—C14	101.9 (2)	C12—C11—H11	115.3
C4—C3—H3	113.0	C2—C11—H11	115.3
C2—C3—H3	113.0	C13—C12—C11	105.2 (3)
C14—C3—H3	113.0	C13—C12—H12	127.4
O2—C4—N1	124.4 (2)	C11—C12—H12	127.4
O2—C4—C3	126.8 (2)	C12—C13—C14	106.3 (3)
N1—C4—C3	108.8 (2)	C12—C13—H13	126.9
C10—C5—C6	120.3 (2)	C14—C13—H13	126.9
C10—C5—N1	119.4 (2)	O3—C14—C13	101.9 (2)
C6—C5—N1	120.3 (2)	O3—C14—C3	99.7 (2)
C7—C6—C5	119.9 (2)	C13—C14—C3	107.0 (2)
C7—C6—H6	120.1	O3—C14—H14	115.4
C5—C6—H6	120.1	C13—C14—H14	115.4
C8—C7—C6	119.3 (2)	C3—C14—H14	115.4
			
C4—N1—C1—O1	179.0 (3)	C5—C6—C7—C8	0.8 (4)
C5—N1—C1—O1	−4.8 (4)	C6—C7—C8—C9	−0.8 (4)
C4—N1—C1—C2	−1.9 (3)	C6—C7—C8—Cl1	−179.9 (2)
C5—N1—C1—C2	174.3 (2)	C7—C8—C9—C10	0.4 (4)
O1—C1—C2—C3	−176.4 (3)	Cl1—C8—C9—C10	179.5 (2)
N1—C1—C2—C3	4.5 (3)	C8—C9—C10—C5	0.2 (4)
O1—C1—C2—C11	75.3 (4)	C6—C5—C10—C9	−0.2 (4)
N1—C1—C2—C11	−103.8 (2)	N1—C5—C10—C9	−178.5 (2)
C1—C2—C3—C4	−5.3 (3)	C14—O3—C11—C12	−49.2 (2)
C11—C2—C3—C4	111.7 (2)	C14—O3—C11—C2	59.1 (2)
C1—C2—C3—C14	−120.4 (2)	C1—C2—C11—O3	78.3 (2)
C11—C2—C3—C14	−3.5 (2)	C3—C2—C11—O3	−33.5 (2)
C1—N1—C4—O2	176.7 (3)	C1—C2—C11—C12	−175.1 (2)
C5—N1—C4—O2	0.4 (4)	C3—C2—C11—C12	73.1 (2)
C1—N1—C4—C3	−1.6 (3)	O3—C11—C12—C13	31.7 (3)
C5—N1—C4—C3	−177.9 (2)	C2—C11—C12—C13	−74.2 (3)
C2—C3—C4—O2	−173.9 (3)	C11—C12—C13—C14	0.3 (3)
C14—C3—C4—O2	−65.0 (4)	C11—O3—C14—C13	49.1 (2)
C2—C3—C4—N1	4.3 (3)	C11—O3—C14—C3	−60.8 (2)
C14—C3—C4—N1	113.3 (2)	C12—C13—C14—O3	−31.9 (3)
C4—N1—C5—C10	128.6 (3)	C12—C13—C14—C3	72.3 (3)
C1—N1—C5—C10	−47.3 (4)	C4—C3—C14—O3	−71.6 (2)
C4—N1—C5—C6	−49.7 (4)	C2—C3—C14—O3	39.1 (2)
C1—N1—C5—C6	134.5 (3)	C4—C3—C14—C13	−177.4 (2)
C10—C5—C6—C7	−0.2 (4)	C2—C3—C14—C13	−66.7 (3)
N1—C5—C6—C7	178.0 (2)		
